# Experimental study and analysis of CO_2_ and SO_2_ absorption in various water-based nanofluids by response surface methodology

**DOI:** 10.1038/s41598-024-56181-4

**Published:** 2024-07-24

**Authors:** Soroush Karamian, Feridun Esmaeilzadeh, Dariush Mowla, Seyyed Hamid Esmaeili-Faraj, Alireza Arjomand

**Affiliations:** 1 Environmental Research Center in Petroleum and Petrochemical Industries, School of Chemical and Petroleum Engineering, Shiraz University, Shiraz, 7134851154 Iran; 2 Department of Material and Chemical Engineering, Shahrood University of Technology, Shahrood, 3619995161 Iran

**Keywords:** Absorption, Nanofluid, Box-Behnken design, Acidic gases, Design of experiments, Nanoscience and technology, Engineering, Chemical engineering

## Abstract

The absorption of acidic gases in the oil and gas industries is important due to their toxicity and corrosive effects. Recently, the application of nanofluids based on aqueous or organic solvents as absorbents has been examined by a variety of researchers. In this study, a single bubble column was exploited to study the effect of water-based nanofluids on the absorption processes of SO_2_ and CO_2_ using response surface methodology (RSM) based on Box-Behnken three-level experiment design. With this in mind, CO_2_ and SO_2_ are separately injected at the bottom of a bubble column filled with one of the nanofluids: Al_2_O_3_-water, SiO_2_-water, or ZnO-water for each experiment. Then, the rate of SO_2_ or CO_2_ absorption in the nanofluids has been elucidated. The effect of important parameters including the weight fraction of the nanoparticles (NPs) (0.01, 0.055, and 0.1 wt.%), gas–liquid contact time (150, 300, and 450 s), and the diameter of nozzle for gas injection (0.46, 0.57, and 0.68 mm) have been studied. Results revealed that the maximum molar flux of both gases was observed in the ZnO-water nanofluid, followed by the SiO_2_-water nanofluid. In addition, increasing the nanoparticle mass fraction and the bubble size causes the molar flux to rise. However, increasing the gas–liquid contact time causes the molar flux of the mentioned gases to decrease. Finally, a set of the accurate equations has been proposed to predict the molar flux of SO_2_ and CO_2_ in the various nanofluids assessed in this work.

## Introduction

The presence of NPs has a significant effect on gas absorption into liquids^1,2^. This effect depends on the material and interaction of gas and NPs^3–5^, as well as the size^6^, concentration^7–10^, and type^11^ of the particles. There are a few reports for investigation of the influence of the above parameters on mass transfer into the nanofluids^12–14^. Ashrafmansouri et al. reviewed previous researches to highlight the impacts of NPs in mass transfer processes^15^.

Researchers have proposed two main mechanisms for mass transfer enhancement by NPs^16^. The first mechanism is called the grazing or shuttle effect, which was presented by Zhao et al.^16^, and defined as “the adsorption of gas molecules on the NPs surfaces at the bubble interface and then release from the NPs surfaces into the fluid bulk”^13^. The second proposed mechanism is hydrodynamic effects, which were adopted by several researchers^16–18^. They believed that the Brownian motion of NPs induces micro-convections in nanofluids and so enhances the rate of mass transfer^16–18^.

Karamian et al. have studied the impact of different water-based nanofluids with Al_2_O_3_, Fe_2_O_3_, and or SiO_2_ NPs on acidic gases (SO_2_ and CO_2_) absorption^19^. They concluded that the types of gas molecules and NPs determined the mechanism of mass transfer enhancement by nanofluids^19^. The effects of nanoparticle size on carbon dioxide absorption in SiO_2_-water nanofluid have been studied by Darvanjooghi et al.^6,20^. They used a bubble column with frequent single bubbles raising into the SiO2-water nanofluid with different particle sizes of 10.6, 20, 38.6, and 62 nm^6^. Esmaeili-Faraj et al. used a bubble column for studying H_2_S absorption by employing exfoliated graphene oxide (EGO) as a nanoparticle^11^. They determined that the addition of NPs to water enhanced mass transfer by 40%. Results show that the grazing effect was the reason for mass transfer enhancement in EGO nanofluids^11^. In the other work, they used EGO-water and the synthesized SiO_2_-water nanofluids for the absorption of acidic gases (H_2_S and CO_2_)^13^. They concluded that oxygen group functionalities on the surface of EGO and SiO_2_ NPs adsorbed gas molecules and were the possible reason for mass transfer enhancement by nanofluids^13^.

In addition to the nanoparticle type, size, and concentration, hydrodynamic properties affect the rate of mass transfer in a bubble column. Bubble size and contact time are two main properties that have a direct effect on the mass transfer rate. Kim et al.^12^ investigated the rate of mass transfer and hydrodynamics of a gas–liquid system for the absorption of CO_2_ in the presence of SiO_2_ NPs. According to their results, the smaller bubbles have a higher surface area and consequently a higher mass transfer rate^12^. They reported that the amount of absorption enhancement in the nanofluid was 24% more than that in water. They believed that the adsorption of micro-metric gas bubbles on the surface of the NPs was the main reason for that boost. Also, they claimed that the presence of stable NPs in the nanofluid provides additional energy needed to dissolve CO_2_ into solution, as well as increasing the total surface area of the gas bubbles due to their collision with the NPs, which causes them to break down into smaller bubbles. All of these phenomena have an impact on the mass transfer enhancement by nanofluids^12^.

The general rules that can explain the relationship between the amount of gas absorption and the size of the bubbles can be summarized as follows^21–24^:

1. Smaller bubbles have more surface area.
2. According to the Laplace-Young relationship (ΔP = P_in_-P_out_ = 2γ/R), the internal pressure of gas bubbles increases with decreasing size.
3. According to the Kelvin relation $\left(RTln(P/P0\right)=2\gamma V/r)$, solubility increases with decreasing bubble size.

Despite the importance of hydrodynamic parameters in gas absorption studies, researchers have rarely investigated them. Therefore, in this research, the type and concentration of NPs, as well as gas–liquid contact time and gas bubble size as two hydrodynamic variables in the bubble column are assessed. The response surface methodology (RSM) approach has been applied for obtaining the optimum formulation. In this methodology, the Box-Behnken design (BBD) approach has been applied to the design of the experiments to obtain the optimum formulation. The effects of the above parameters on mass transfer flux have been studied separately.

## Methods and materials

### Materials

Al_2_O_3_ NPs with a purity of 99.98 wt.% and ZnO NPs with a purity of 99.99 wt.% were purchased from the U.S. Nano Company, United State. SiO_2_ NPs were synthesized according to the method explained in S.H. Esmaeili-Faraj and M. Nasr Esfahany^13^. Table S-1 presents the properties of NPs used in this work. TEM (PHILIPS CM120, USA) and SEM (PHILIPS XL-30 ESEM, USA) images of the Al_2_O_3_, ZnO, and SiO_2_ NPs are depicted in Fig. S-1 in the supplementary. Results of the X-Ray diffraction (XRD, Philips model PW3710 (Amsterdam, Netherlands)) analyses for the Al_2_O_3_ and ZnO NPs, as well as, the synthesized SiO_2_ NPs are shown in Fig. S-2 in the supplementary.

In order to measure the quantity of CO_2_ and SO_2_ dissolved in nanofluids by titration, NaOH pellets (99.99 wt.%) and HCl with a purity of 37 vol.% were purchased from Merck Company, Germany. The phenolphthalein and methyl orange, as indicators in titration progress^25^, were obtained from Merck Company, Germany. Deionized water was used as the base fluid. All the materials were used as received without any further treatment.

### Nanofluids preparation

The NPs were first grinded by a ball-mill device for about 4 h in order to break up the agglomerated NPs into the primary particles. An amount of 5 g for each of the NPs, including SiO_2_, Al_2_O_3_, or Fe_2_O_3_ was then suspended in 1000 mL deionized water by mixing the solution with 800 rpm for 5 h, which is equivalent to 5000 mg/L or 0.5 wt.%. Afterwards, the NPs were dispersed in the base fluid by using an ultrasonic homogenizer (QSONICA-Q700 ultrasonic generator, USA) under three sequences of 20 min with an amplitude of 70% and a cycle time of 0.5 s. Finally, the prepared suspensions were diluted with deionized water to provide different nanoparticle concentrations of 0.01, 0.055, and 0.1wt. %. Figure S-3 shows the nanofluids with 0.1 wt.% during 24-hour time. According to the figure, all nanofluids have high stability. Moreover, Zeta potential test can be done to determine the stability of nanoparticles in base fluid. If the zeta potential is less than − 45 mV, the nanofluid is stable. Zeta potential values are − 100.2 mV for SiO_2_ and − 97.8 mV Al_2_O_3_/water nanofluids. Zeta potential for ZnO/water nanofluid is − 89.3 mV. According to these values, it is clear that nanofluids have high stability.

### Experimental set-up

**Figure 1:**
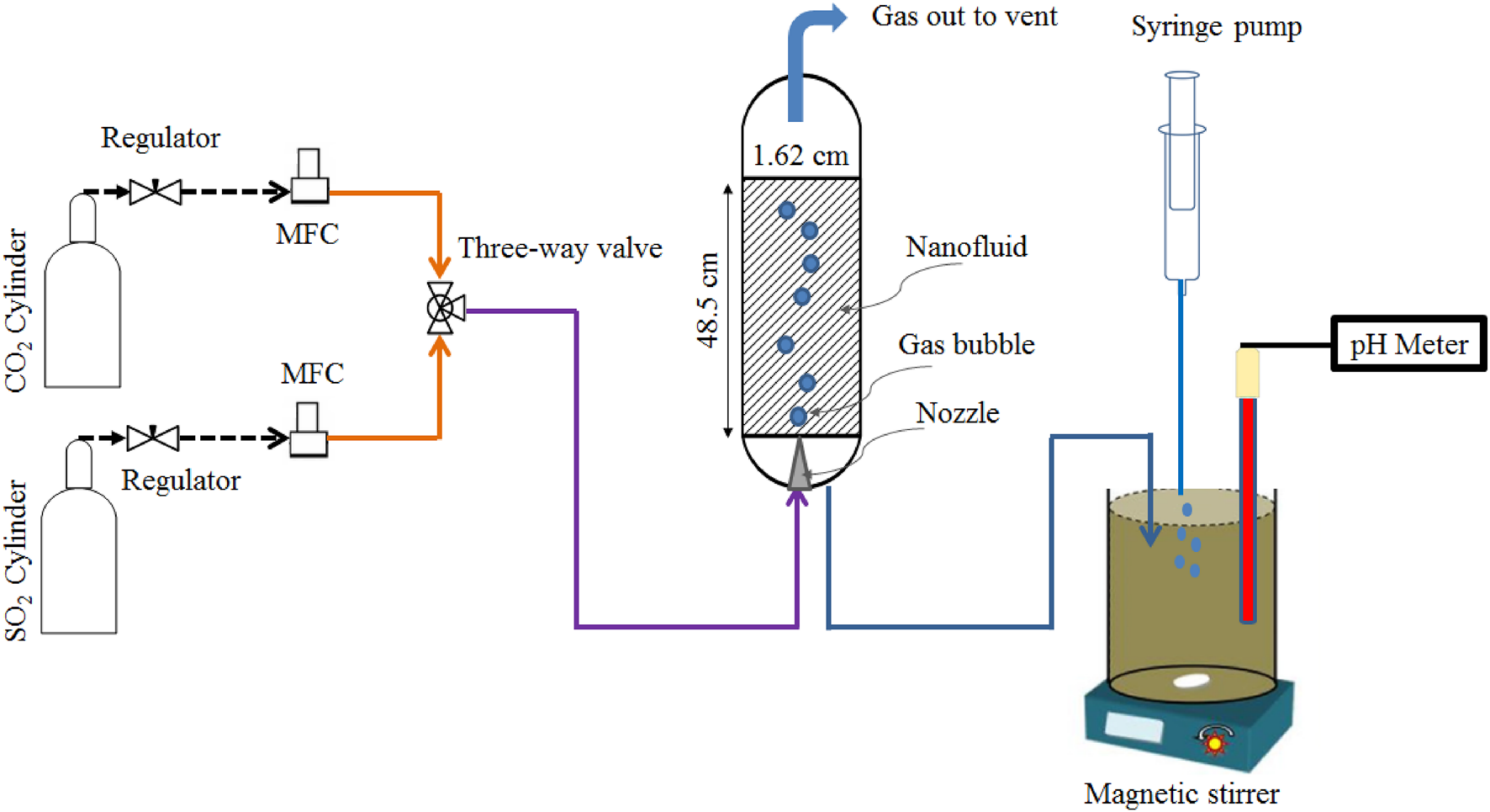
Schematic view of the experimental set-up.

The main part of the experimental set-up is made up of a semi-batch bubble column absorber containing 100 mL of the aforementioned nanofluids. Different volumes of CO_2_ and SO_2_ at a constant flow rate of 2 mL/min with three different contact times of 150, 300, and 450 s were injected singly into the nanofluids kept in the absorption column. Figure 1 shows a schematic diagram of the bubble column absorber with 1 m height and 16.2 mm diameter. The material of the bubble column is poly-methyl-meta-acrylate (PMMA). A pressure/flow controller (Brooks Instrument 1-888-554-flow, USA) was used to control the flow rate of the gas. Finally, the concentration of CO_2_ or SO_2_ in the nanofluids was measured using the reverse titration method, explained in detail in our previous work^19^. Three nozzles with different orifice diameters of 0.46, 0.57, and 0.68 mm were exploited to adjust the diameter of the gas bubbles flowing into the nanofluids from the bottom to the top of the column.

### Design of experiments

In the study of carbon dioxide and sulfur dioxide uptake in various water-based nanofluids with Al_2_O_3_, SiO_2_, and ZnO NPs, three parameters can be considered as independent parameters^13,19^: weight fraction of NPs, gas injection nozzle size, and mass transfer duration. It should be noted that the last parameter corresponds to the gas–liquid contact time. To investigate the effect of each of the aforementioned independent parameters on the mass transfer rate in different nanofluids, as well as the interaction between the parameters, the RSM based on the BBD method was applied. A detailed description of the RSM and BBD methods is reported in the literature^26,27^. To this end, three levels for each factor have been selected, as shown in Table 1. According to the BBD method, 15 runs are required for each of the nanofluids. The BBD is a good design of experiment for the RSM because it allows to estimate the parameters of a quadratic model^26,27^. The Results of the BBD method are explained in the next section.

**Table 1:** Effective variables and their working ranges for Box-Behnken design.

Factors	Lower level (− 1)	Center point (0)	Higher level (+ 1)
A: nozzle size (*d*_*o*_), mm	0.46	0.57	0.68
B: NPs fraction ($\omega$), wt.%	0.01	0.055	0.10
C: contact time ($\tau$), s	150	300	450

## Results and discussion

### Bubble size measurement

In this work, the molar flux of the absorbed gases was calculated using gas concentration in the nanofluids according to the following equation^6,11,13,19^.

1 $${{\text{N}}}_{{\text{ave}},\mathrm{ R}{{\text{O}}}_{2} }=\frac{{{\text{C}}}_{{{\text{RO}}}_{2} }\times {\text{V}}}{(4\uppi {{\text{r}}}_{0}^{2}{\text{n}})\times (\uptau )}\times {10}^{-6}.$$

Here, ${{\text{N}}}_{{\text{ave}},{{\text{RO}}}_{2}}$ is the average molar flux transferred from pure gas, including CO_2_ or SO_2_ into the liquid phase (mol/m^2^ s). ${\varvec{\uptau}}$ is the total gas–liquid contact time of bubbles passing through the nanofluids (s). **n** is the total number of bubbles passing through the nanofluids within the absorber column, and ${{\text{r}}}_{0}$ is the average bubble’s radius. Also, one of the striking parameters affecting the amount of absorption of CO_2_ or SO_2_ in the assessed nanofluids is the gas bubble size gradient along the vertical axis of the column. This size is a function of nozzle diameter. For this reason, three nozzles with different diameters were employed to examine the effect of average gas bubble diameter on the performance of the absorber column. Due to the mass transfer from the gas bubble to the nanofluid, along the vertical axis of the tower, the volume of the gas bubble decreases and consequently the diameter of the bubble along the tower decreases. A preliminary experiment is the measurement of bubble diameter along the column using three nozzles with different orifice sizes. Corresponding photos for this experiment are depicted in Fig. S-4 in the supplementary. The mentioned experiments were fulfilled according to the procedure explained in section "Design of experiments". Due to the low concentration of the NPs in the nanofluids, the type, and concentration of the NPs have no sensible effect on the size of the gas bubbles in the absorption column. According to Fig. S-4 (in the supplementary), the bubble diameter at the top and the bottom of the column are given in Table S-2. As can be seen, the diameter of the gas bubble decreases with increasing the height of the column due to mass transfer from the gas phase to the liquid phase. The average diameters of bubbles along to column are respectively 2.5, 2.9, and 3.3 mm for three nozzles with 0.46, 0.57, and 0.68 mm diameters.

The theoretical number of bubbles is obtained by dividing the total volume of injected gas by the volume of each bubble:

2 $$n=\frac{{V}_{g}}{\frac{1}{6}\pi {d}_{0}^{3}}.$$

The volume of injected gas for the contact time of 150, 300 and 450 s were 5, 10 and 15 ml, respectively. The theoretical number of bubbles were verified by slow motion filming and counting the number of bubbles during a minute. Results show that relative error of the theoretical number of bubbles obtained by Eq. (2) is lower than 12%. In Table S-4 the theoretical number of bubbles are presented.

### Absorption in base fluid (DM water)

In order to be able to compare the absorption of gases in nanofluids and determine the improvement of gas absorption, in this section, the absorption of carbon dioxide and sulfur dioxide gases in demineralized water (DM water) has been investigated. Figure S-5 shows the molar flux values of CO_2_ and SO_2_ for the diameter of different gas injection nozzles according to contact time. Also, an exponential curve has been fitted to the points obtained from the experiments in these graphs, and the equations resulting from the fitting are shown in Table 2. As can be seen, the exponential equations are well fitted with the experimental data. This exponential equation is in agreement with the following equation presented by Esmaeili-Faraj et al.^11^:

**Table 2:** Correlation for molar flux of CO_2_ and SO_2_ in DM water.

Gas type	Nozzle diameter, mm	Correlation	R-square
CO_2_	0.46	${N}_{C{O}_{2}}=0.0001{e}^{-0.004\tau }$	0.9166
	0.57	${N}_{C{O}_{2}}=0.0004{e}^{-0.004\tau }$	0.9845
	0.68	${N}_{C{O}_{2}}=0.0004{e}^{-0.004\tau }$	0.9999
SO_2_	0.46	${N}_{S{O}_{2}}=4\times {10}^{-5}{e}^{-0.001\tau }$	0.9063
	0.57	${N}_{S{O}_{2}}=8\times {10}^{-5}{e}^{-0.003\tau }$	0.9302
	0.68	${N}_{S{O}_{2}}=3\times {10}^{-4}{e}^{-0.004\tau }$	0.9402

3 $${N}_{A,av}=m{e}^{-K\tau }.$$

### Implementation of the BBD method

To investigate the absorption of CO_2_/SO_2_ gases by nanofluids of SiO_2_-water, Al_2_O_3_-water, and ZnO-water, the BBD method was carried out with three factors: nozzle size, nanoparticle mass fraction, and total gas–liquid contact time. According to the BBD method, 15 experiments should be performed for each gas and each nanofluid. In summary, six sets of 15 experiments were fulfilled. The results of these experiments for CO_2_ and SO_2_ gases are presented in two separate parts below.

#### CO_2_ absorption results

Table S-3 shows the results of the average molar flux of CO_2_ (${{\text{N}}}_{{\text{ave}},\mathrm{ C}{{\text{O}}}_{2}}$) and the amount of CO_2_ absorbed by the nanofluids according to the BBD of the experiment. The suitable equation to predict the responses in BBD is a quadratic equation^26,27^. Based on a quadratic equation, the analysis of variance (ANOVA) for the molar flux of CO_2_ into the nanofluids is shown in Table 3.

**Table 3:** Results of ANOVA for the molar flux of CO_2_ into the nanofluids.

Source	CO_2_			SO_2_		
	Al_2_O_3_-W NF	SiO_2_-W NF	ZnO-W NF	Al_2_O_3_-W NF	SiO_2_-W NF	ZnO-W NF
	*p*-value	*p*-value	*p*-value	*p*-value	*p*-value	*p*-value
Model	0.0008	0.0035	0.0005	0.0088	0.0094	0.0044
A: (*d*_*o*_)	0.0011	0.0186	0.0012	0.0043	0.0024	0.0273
B: ($\omega$)	0.1271	0.7602	0.2750	0.0882	0.2469	0.8089
C: ($\tau$)	< 0.0001	0.0003	< 0.0001	0.0017	0.0018	0.0002
AB	0.4965	0.2317	0.7568	0.6236	0.6105	0.4957
AC	0.0062	0.2317	0.0057	0.0225	0.0249	0.1220
BC	0.0614	0.8670	0.1767	0.4025	0.1973	0.3251
A^2^	0.1913	0.0155	0.1051	0.0521	0.1693	0.0661
B^2^	0.6695	0.6860	0.5787	0.9887	0.9968	0.2578
C^2^	0.0039	0.0035	0.0015	0.0147	0.0280	0.0102
Model type	Quadratic	Quadratic	Quadratic	Quadratic	Quadratic	Quadratic
R-square	0.9819	0.9664	0.9847	0.9508	0.9496	0.9632
Adjusted R^2^	0.9493	0.9060	0.9572	0.8623	0.8587	0.8970

In the ANOVA approach, the ***p***-value is an important factor. When the ***p***-value is less than 0.05, it means the model/parameter is significant^27^. The larger ***p***-values indicate that the model/parameter is insignificant. According to the results of Table 3, for all three types of nanofluids, the model ***p***-values are less than 0.05, and therefore all three models are significant. In addition, the parameters A, C, AC, and C^2^ for Al_2_O_3_-water nanofluid, A, C, A^2^, and C^2^ for SiO_2_-water nanofluid, and A, C, AC, and C^2^ for ZnO-water nanofluid are significant parameters. Moreover, the R-square of the quadratic equations obtained for the Al_2_O_3_-water, SiO_2_-water, and ZnO-water nanofluids are 0.9819, 0.9675, and 0.9554, respectively. The other factors of ANOVA that are presented in Table 3 indicate the high accuracy and quality of the model.

#### SO_2_ absorption results

Similar to the previous section, results of the average molar flux of SO_2_ (${{\text{N}}}_{{\text{ave}},\mathrm{ S}{{\text{O}}}_{2}}$) and the amount of SO_2_ absorbed by the nanofluids, according to the BBD of the experiment, are reported in Table S-4. In addition, Table 3 shows the ANOVA for the molar flux of SO_2_ into the nanofluids based on a quadratic equation.

According to the results of Table 3, for all three types of nanofluids, the model ***p***-values are less than 0.05, so all three models are significant. In addition, the parameters A, C, AC and C^2^ for Al_2_O_3_-water nanofluid, A, C, AC, and C^2^ for SiO_2_-water nanofluid, and A, C, and C^2^ for ZnO-water nanofluid are significant parameters. Moreover, the R-square of the quadratic equations obtained for the Al_2_O_3_-water, SiO_2_-water, and ZnO-water nanofluids are 0.9015, 0.9496, and 0.9632, respectively. So, according to the ANOVA results, it is concluded that the quadratic model has high accuracy.

Based on the results of the last two sections, two sets of quadratic equations can be obtained to predict the mass transfer fluxes of CO_2_ and SO_2_ according to Table 4. In these equations, the molar flux is expressed in terms of three variables: nozzle diameter (*d*_*o*_), the weight fraction of NPs ($\omega$), and gas–liquid contact time ($\tau$). These equations are different depending on the type of gas and the type of nanofluid. Needless to say, these equations are only applicable to the bubble columns.

**Table 4:** The obtained quadratic equations for the prediction of molar fluxes of CO_2_ and SO_2_ in the nanofluids.

Gas type	Nanofluid type	Obtained equation based on BBD method
CO_2_	Al_2_O_3_-W NF	${N}_{C{O}_{2}}=-0.001058 + 0.004208 * {d}_{o} + 0.000404 * \omega -4.03699e-07 * \tau + 0.002346 * {d}_{o}* \omega -4.3526e-06 *{d}_{o} * \tau -5.64122e-06 * \omega * \tau -0.002059 * {d}_{o}^{2} + 0.00369 * {\omega }^{2} + 3.71877e-09 * {\tau }^{2}$
	SiO_2_-W NF	${N}_{C{O}_{2}}=-0.002002 + 0.008836 * {d}_{o} + 0.003092 * \omega -2.95113e-06 * \tau -0.005848 * {d}_{o} *\omega -1.75455e-06 * {d}_{o} * \tau -5.55556e-07 * \omega * \tau -0.006596 * {d}_{o}^{2} +0.004687 * {\omega }^{2} + 5.09296e-09 * {\tau }^{2}$
	ZnO-W NF	${N}_{C{O}_{2}}=-0.001418 + 0.00615 * {d}_{o} + 0.000348 * \omega -1.35e-06 * \tau +0.001286 * {d}_{o} *\omega -5.44636e-06 * {d}_{o} * \tau -4.53015e-06 * \omega * \tau -0.003306 * {d}_{o}^{2} +0.005934 * {\omega }^{2} + 5.6326e-09 * {\tau }^{2}$
SO_2_	Al_2_O_3_-W NF	${N}_{S{O}_{2}}=+0.000617- 0.00225 * {d}_{o} + 0.0002 * \omega -1.41394e-07 * \tau +0.001452 * {d}_{o} *\omega -2.71407e-06 * {d}_{o} * \tau -1.86292e-06 * \omega * \tau +0.003002 * {d}_{o}^{2}-0.000105 * {\omega }^{2} + 2.32481e-09 * {\tau }^{2}$
	SiO_2_-W NF	${N}_{S{O}_{2}}=0.000095- 0.000649 * {d}_{o} + 0.001685 * \omega +8.98625e-08 * \tau -0.001237 * {d}_{o} *\omega -2.16577e-06 * {d}_{o} * \tau -2.4839e-06 * \omega * \tau +0.001558 * {d}_{o}^{2}-0.000025 * {\omega }^{2} +1.599e-09 * {\tau }^{2}$
	ZnO-W NF	${N}_{S{O}_{2}}=-0.001653+ 0.007255 * {d}_{o} + 0.005006 * \omega -2.07147e-06 * \tau -0.003691 * {d}_{o} *\omega -2.80421e-06 * {d}_{o} * \tau -4.02089e-06 * \omega * \tau -0.005015 * {d}_{o}^{2}-0.016324 * {\omega }^{2} + 4.61985e-09 * {\tau }^{2}$

To illustrate the accuracy of the obtained equations with the results of experimental tests, the graphs of the predicted versus actual values for the molar flux of each gas in different nanofluids are shown in Fig. S-6. The solid line represents the graph of *y* = *x,* and the points shown in these figures illustrate the difference between the predicted values of the mass transfer flux using the quadratic equations reported in Table 4 and the measured values for the molar flux that were reported in Tables S-3 and S-4. If the predicted and actual values are the same, they will fit on the solid line and the greater differences lead to more distance from the solid line. It can be concluded from the diagrams in Fig. S-6 that the accuracy of the resulting equations is adequate for all of the nanofluids and both CO_2_ and SO_2_ gases.

### Effect of factors on molar flux

**Figure 2:**
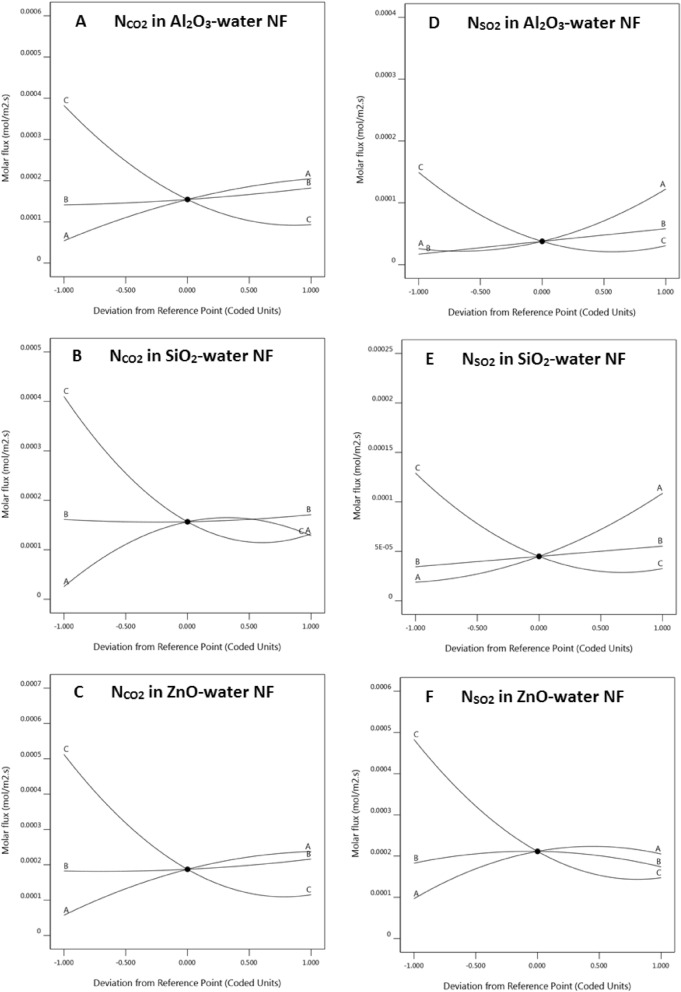
The molar flux of CO_2_ (**A** to **C**) and SO_2_ (**D** to **F**) into the various nanofluids versus three factors: Nozzle size ((**A**) curve), nanoparticle fraction ((**B**) curve), and contact time ((**C**) curve).

In this study, in addition to gas type and nanoparticle type, the effect of three basic factors—nozzle diameter, mass fraction of NPs, and gas–liquid contact time-on mass transfer flux was evaluated. In this section, the effect of each of the above factors on the mass transfer flux is analyzed in detail. The basis of this assessment is available in Fig. 2. In the Fig. 2, diagrams A to C, are related to the molar flux of CO_2_ in the nanofluids, and the D to F diagrams are related to the molar flux of SO_2_ in the nanofluids. The horizontal axis of these diagrams is each of the three factors of nozzle size, nanoparticle fraction, and contact time, which are coded by A, B, and C, respectively. The points highlighted in these diagrams correspond to the center point according to Table 1. On the horizontal axis, the coded units are in accordance with Table 1.

#### Effect of gas and nanoparticle type

According to the previous article^19^, the gas type and nanoparticle type have an important role in mass transfer by nanofluids. The interaction of gas molecules and NPs determines the mechanism of enhancement of mass transfer. According to Fig. 2, the absorption of SO_2_ in Al_2_O_3_-water and SiO_2_-water nanofluids is much lower than that of CO_2_ in these nanofluids. However, for the ZnO nanofluid, the molar flux of absorption of both gases is almost the same. Importantly, both CO_2_ and SO_2_ absorption in ZnO nanofluid is faster than in the other nanofluids, and in general, it can be written as follows:

4 $$\left({N}_{R{O}_{2}} \,\,in\,\, ZnO NF\right)>\left({N}_{R{O}_{2}} \,\,in \,\,Si{O}_{2} NF\right)>\left({N}_{R{O}_{2}}\,\, in\,\, A{l}_{2}{O}_{3} NF\right).$$

This observation has also been reported in other researches^28,29^. In most cases, Al_2_O_3_ has much lower absorption than other nanoparticles, and ZnO has a higher absorption effect due to its higher activity and its physical and chemical properties. This observation continues to prove that the uptake by nanoparticles is due to the grazing effect that was previously explained by Esmaeili-Faraj et al.^13^.

#### Effect of bubble diameter

The diameter of the gas bubbles, as previously stated, is directly proportional to the size of the bubble injector (nozzle size)^21,22^. When the size of the bubbles is smaller, it is expected that the mass transfer area will increase and, therefore, the mass transfer flux will decrease. By checking parameter A in all the diagrams of Fig. 2, such behavior is observed. However, in diagrams B and F, the curves for the nozzle size (A) have a slight maximum point while the mass transfer fluxes at the 0 and + 1 points (corresponding to 0.57 and 0.68 mm, respectively) are not different. Therefore, the size of the nozzle diameter has two different effects on the mass transfer flux. On the one hand, reducing the diameter of the nozzle leads to a decrease in the bubble area, but on the other hand, it will increase the number of bubbles and also increase the gas–liquid contact time, so in opposition to these two effects, the mass transfer flux can increase or decrease.

#### Effect of nanoparticle fraction

According to the previous studies^11,13^, the effect of the presence of NPs partly improves the mass transfer in the nanofluids, and after reaching a certain amount, because of unknown reasons, the mass transfer decreases. Esmaeili-Faraj et al. have suggested that the NPs in the vicinity of the bubbles prevent the movement of NPs that carry gas molecules into the liquid bulk^11^. Also, for each nanofluid, the fraction of NPs with the highest adsorption enhancement is different, so that for some NPs, even up to a few percent by weight of NPs, the mass transfer can be improved. According to the results of the previous article^19^, SiO_2_-water and Al_2_O_3_-water had the highest adsorption at a fraction of 0.1 wt.%. So, in this study, the nanoparticle fraction was considered to be 0.01 to 0.1 wt.%. In Fig. 2, it is observed that for these two nanofluids, increasing the fraction of NPs continuously increases the mass transfer flux. Whereas for the ZnO-water nanofluid, the maximum flux is seen at the central point. Therefore, the optimum nanoparticle fraction for ZnO-water nanofluid will be 0.055 wt.%, and for the other nanofluids is 0.1 wt.%.

#### Effect of gas–liquid contact time

Increasing the gas–liquid contact time has always increased the amount of gas absorption until the two phases reach equilibrium (at long times)^30^. As the two phases approach equilibrium, the mass transfer flux tends to zero. As a result, it is important to know the contact time where the mass transfer flux is not negligible. For this reason, the contact time (τ) of the two phases was considered one of the important factors affecting the mass transfer. As shown in Fig. 2, in all cases at lower contact times, where τ = 150 s, there is the highest mass transfer flux. Nevertheless, with increasing contact time to 300 s, the flux decreases sharply and then decreases slowly with increasing contact time to 450 s. According to these diagrams, we can say that the best contact time for gas and liquid phases in all cases is in the range of 200 s to 250 s. At higher contact times, the mass transfer flux will be very low, which is not desirable. In the other hand, at lower contact times, the gas flow rate (in this study, the volume of injected gas) will be too little. Therefore, the median of these values has suitable effects. It is mentioned that in this study, the injectable gas flow to the tower has always been constant and equal to 2 ml/min. Since the volume flow rate will be obtained from the division of gas volume to the gas–liquid contact time, so the higher the gas volume, cause to the higher the contact time, and the lower the gas volume, the lower the contact time.

#### Effect of interaction of parameters

**Figure 3:**
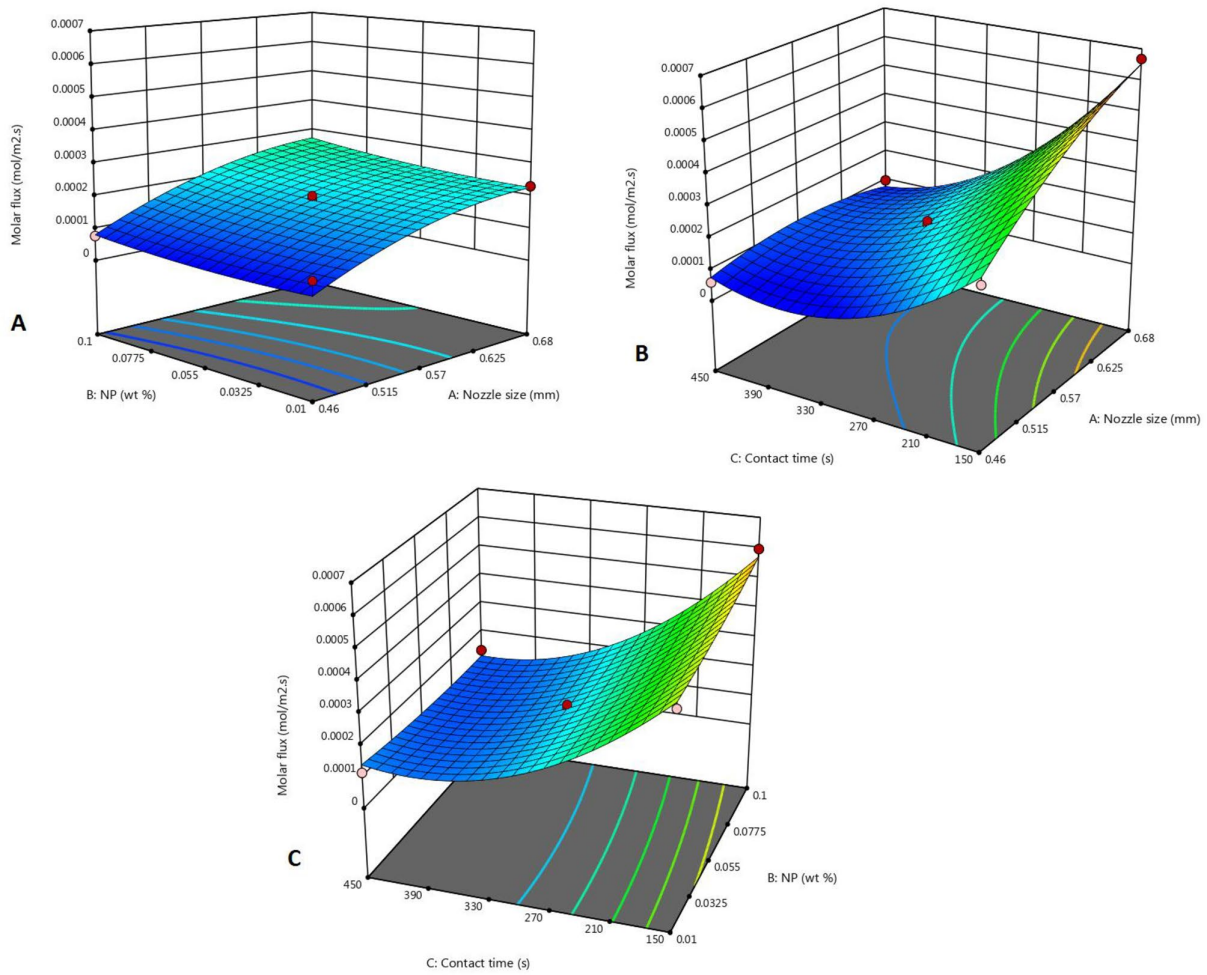
Effect of parameters on molar flux of CO_2_ in ZnO-water nanofluid: (**A**) *d*_*o*_ and $\omega$, (**B**) *d*_*o*_ and $\tau$, (**C**) $\omega$ and $\tau$.

**Figure 4:**
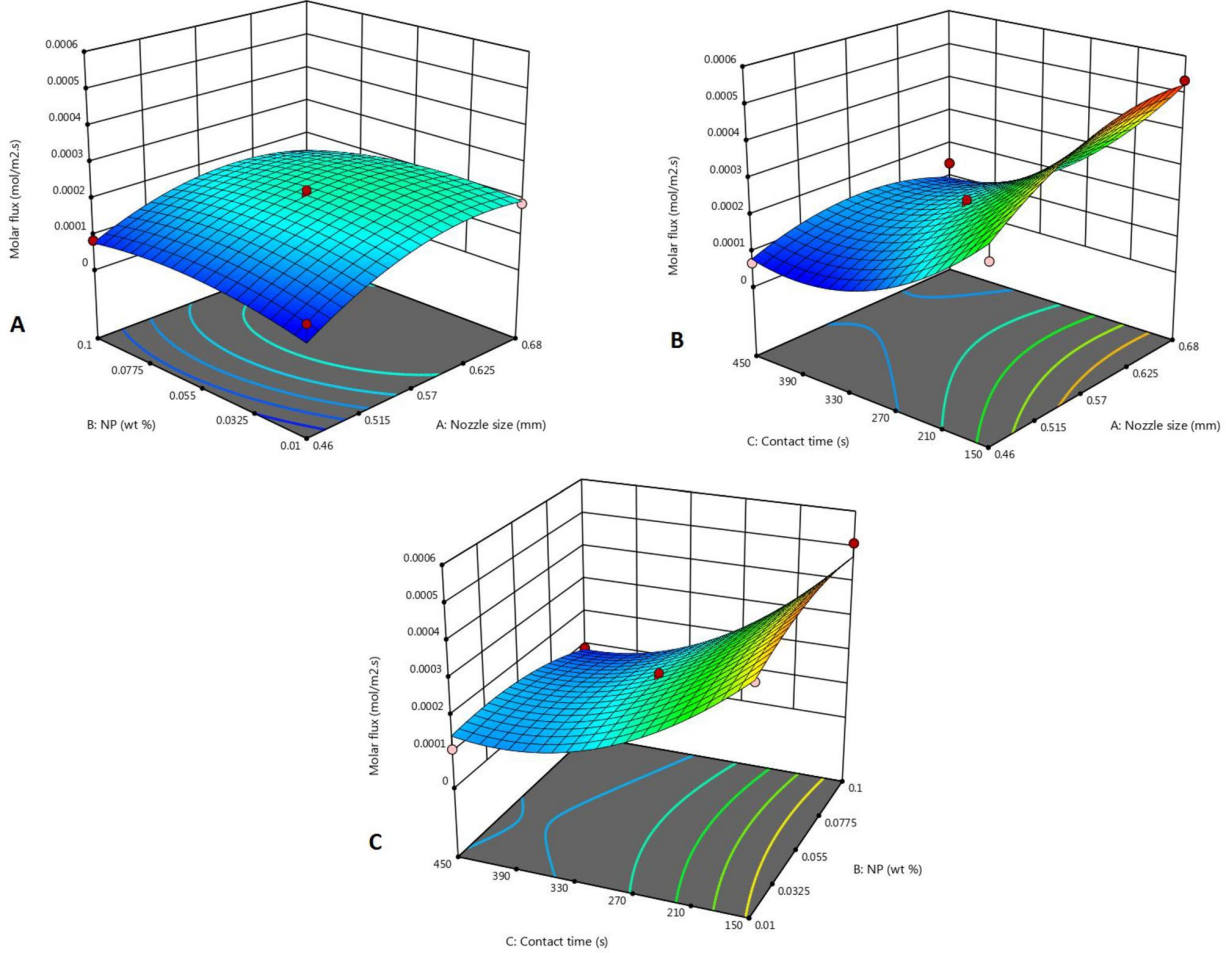
Effect of parameters on molar flux of SO_2_ in ZnO-water nanofluid: (**A**) *d*_*o*_ and $\omega$, (**B**) *d*_*o*_ and $\tau$, (**C**) $\omega$ and $\tau$.

In this section, the interaction of the parameters has been studied. 3D-plots for CO_2_ and SO_2_ molar flux versus the above-mentioned parameters for ZnO-water nanofluid as an example are, respectively, presented in Figs. 3 and 4. The other 3D-plots for Al_2_O_3_-water and SiO_2_-water nanofluids are presented in the supplementary (Figs. S-7 and S-9 for CO_2_ and Figs. S-8 and S-10 for SO_2_, respectively).

According to Fig. 3A, in all weight fractions of nanoparticles, the molar flux increases with increasing nozzle size, so these two parameters do not interact. According to Fig. 3B, in low contact times e.g. 150 s, increasing the diameter of the nozzle uniformly increases the molar flux, and in higher contact times e.g. 450 s, with increasing nozzle size, a small increase in molar flux is observed, then it decreases. The reason for this, as stated earlier, is that on the one hand, reducing the diameter of the nozzle leads to a decrease in the area of each bubble, but on the other hand, it will lead to an increase in the number of bubbles and an increase in the gas–liquid contact time. The two parameters of contact time and weight fraction of nanoparticles also do not interact with each other, which is evident in Fig. 3C. The same behavior can be seen for the absorption of sulfur dioxide shown in Fig. 4.

#### Optimum parameters

One of the capabilities of the RSM is to find optimal conditions. This feature is available in the Design Expert software. Therefore, the optimal conditions for having the highest CO_2_ and SO_2_ mass transfer flux are shown in Table 5.

**Table 5:** Optimum conditions for CO_2_ and SO_2_ absorption by the nanofluids.

Gas type	Nanofluid type	Optimum parameters			Molar flux, (mol/m^2^.s)
		A: nozzle size (mm)	B: NP fraction (wt.%)	C: contact time (s)	
CO_2_	Al_2_O_3_-water	0.68	0.09	152	0.000517877
	SiO_2_-water	0.65	0.01	150	0.000467000
	ZnO-water	0.68	0.072	150	0.000668229
SO_2_	Al_2_O_3_-water	0.68	0.091	150	0.000318797
	SiO_2_-water	0.68	0.100	150	0.000246941
	ZnO-water	0.66	0.06	150	0.000534583

## Conclusion

Acid gases are commonly present in gas mixtures in oil, gas and petrochemical industries. Acidic gases, in particular, are significant due to their corrosive, toxic, and greenhouse effects. Separation of these components is done by conventional methods of mass transfer, including scrubbing processes. Recently, researchers have suggested the application of nanofluids based on aqueous or organic solvents as absorbents. In this study, a single bubble column was used to study the effect of water-based nanofluids with Al_2_O_3_, SiO_2_, and ZnO NPs on the absorption of SO_2_ and CO_2_. In the experiments, SO_2_ and CO_2_ were separately injected into the end of a bubble column, each filled with one nanofluid, and the molar flux was measured. To investigate the effect of important parameters on the mass transfer process in the single bubble column, a response surface methodology design based on the Box-Behnken three-level method was used. Three parameters were studied in these experiments: the NPs fraction (NP%), the gas–liquid contact time (τ), and the nozzle size of the gas injection, as well as, the interaction of gas type and nanoparticle type. A set of equations for analyzing the mass transfer flux and the amount of gas absorbed based on these three parameters was then obtained. Finally, it was applied to each nanofluid, and the optimum operating conditions for CO_2_ and SO_2_ gases were determined by designing experiments. It was found that the molar flux of absorption of CO_2_ and SO_2_ gases by the ZnO nanofluid is almost the same, and both CO_2_ and SO_2_ absorption in the ZnO nanofluid is faster than in the other nanofluids. It was shown that the optimal nanoparticle fraction for ZnO-water nanofluid is about 0.06–0.07 wt.%, and approximately 0.1 wt.% for the other nanofluids, and finally, in all cases, the optimal contact time for gas and liquid phases is about 150 s. Comparing of absorption in nanofluids and base fluid shows that SO_2_ and CO_2_ molar flux in nanofluids was intensified up to two-fold more than the base fluid.

### Supplementary Information

Supplementary Information.

## Data Availability

All data generated or analyzed during this study are included in this published article (the supplementary file, Tables S-3 and S-4).
