# Supplementary material for: Experimental study and analysis of CO2 and SO2 absorption in various water-based nanofluids by response surface methodology
Source: Sci Rep. 2024 Jul 24;14:17108. doi: 10.1038/s41598-024-56181-4 (PMC11269607; doi:10.1038/s41598-024-56181-4)
Supplement: Supplementary file 1 — Supplementary Information. [file 41598_2024_56181_MOESM1_ESM.docx]

**Supplementary File for:**

**Experimental Study and Analysis of CO_2_ and SO_2_ Absorption in Various Water-based Nanofluids by Response Surface Methodology**

Soroush Karamian^1^, Feridun Esmaeilzadeh^1,*^, Dariush Mowla^1^, Seyyed Hamid Esmaeili-Faraj^2,*^, Alireza Arjomand^2^

1. Environmental Research Center in Petroleum and Petrochemical Industries, School of Chemical and Petroleum Engineering, Shiraz University, Shiraz 7134851154, Iran.
2. Department of Material and Chemical Engineering, Shahrood University of Technology, Shahrood, 3619995161, Iran.

*Corresponding authors: [h.esmaeili@shahroodut.ac.ir](mailto:h.esmaeili@shahroodut.ac.ir) , esmaeil@shirazu.ac.ir

**Table S-1.** Physical properties of the NPs used in this study.

| **Properties** | **SiO_2_ NPs** | **Al_2_O_3_ NPs** | **ZnO NPs** |
| --- | --- | --- | --- |
| Molecular weight, g/mol | 60.08 | 101.96 | 81.40 |
| Diameter, nm | ~20 | ~20 | ~20 |
| Density, g/cm^3^ | 2.196 | 3.980 | 5.600 |
| Melting point, °C | 1713 | 2054 | 1975 |
| Appearance | White Powder | White Powder | White Powder |

**Table S-2.** The bubble diameter of CO_2_ gas passes into the base fluid (at 25 °C, 1 atm) from the bottom to the top of the absorption column for various nozzle sizes.

| Nozzle size, mm | Bottom | Top | Average bubble diameter, mm |
| --- | --- | --- | --- |
| 0.46 | 3.5 | 1.5 | 2.5 |
| 0.57 | 3.7 | 2.2 | 2.9 |
| 0.68 | 4.2 | 2.5 | 3.3 |

**Table S-3.** Results of the average molar flux and the amounts of CO_2_ absorbed by nanofluids

| **SiO_2_-water NF** | | **ZnO-water NF** | | **Al_2_O_3_-water NF** | | **C:**  $\tau$  **(s)** | **B:**  $\omega$ **(wt.%)** | **A:**  *d_o_* **(mm)** | **Run No.** |
| --- | --- | --- | --- | --- | --- | --- | --- | --- | --- |
| **N_avg×10_^6^**  **mol/m^2^.s** | **C­_Bulk,_**  **mol/m^3^** | **N_avg×10_^6^**  **mol/m^2^.s** | **C­_Bulk,_**  **mol/m^3^** | **N_avg×10_^6^**  **mol/m^2^.s** | **C­_Bulk,_ mol/m^3^** |  |  |  |  |
| 56.83±0.61 | 7.8610 | 59.27±0.64 | 8.1979 | 51.9±0.56 | 7.1872 | 450 | 0.055 | 0.46 | 1 |
| 164.06±2.19 | 5.0535 | 189.58±2.49 | 5.8396 | 156.77±2.11 | 4.8289 | 300 | 0.055 | 0.57 | 2 |
| 466.66±8.91 | 3.5936 | 597.91±11.1 | 4.6043 | 466.66±8.91 | 3.5936 | 150 | 0.100 | 0.57 | 3 |
| 116.67±1.35 | 8.0856 | 123.15±1.42 | 8.5348 | 108.56±1.26 | 7.5241 | 450 | 0.100 | 0.57 | 4 |
| 156.77±2.11 | 4.8289 | 200.52±2.62 | 6.1765 | 167.71±2.24 | 5.1658 | 300 | 0.055 | 0.57 | 5 |
| 162.88±2.37 | 4.3797 | 229.70±3.21 | 6.1765 | 192.12±2.74 | 5.1658 | 300 | 0.010 | 0.68 | 6 |
| 384.23±8.69 | 2.5829 | 668.23±14.1 | 4.4920 | 517.88±11.20 | 3.4813 | 150 | 0.055 | 0.68 | 7 |
| 211.92±3.05 | 3.2567 | 270.37±3.67 | 4.1551 | 204.61±2.98 | 3.1444 | 150 | 0.055 | 0.46 | 8 |
| 437.50±8.43 | 3.3690 | 437.50±8.43 | 3.3690 | 306.25±6.35 | 2.3583 | 150 | 0.010 | 0.57 | 9 |
| 113.23±1.38 | 6.8503 | 94.67±1.18 | 5.7273 | 77.96±0.99 | 4.7166 | 450 | 0.055 | 0.68 | 10 |
| 67.59±0.82 | 4.1551 | 74.90±0.89 | 4.6043 | 58.46±0.74 | 3.5936 | 300 | 0.100 | 0.46 | 11 |
| 102.08±1.19 | 7.0749 | 115.04±1.33 | 7.9733 | 100.46±1.18 | 6.9626 | 450 | 0.010 | 0.57 | 12 |
| 12.79±0.43 | 0.7861 | 100.48±1.14 | 6.1765 | 84.03±0.98 | 5.1658 | 300 | 0.010 | 0.46 | 13 |
| 149.48±2.03 | 4.6043 | 171.35±2.28 | 5.2781 | 138.54±1.90 | 4.2674 | 300 | 0.055 | 0.57 | 14 |
| 162.88±2.37 | 4.3797 | 250.59±3.48 | 6.7380 | 212.00±3.00 | 5.7273 | 300 | 0.100 | 0.68 | 15 |

**Table S-4.** Results of average molar flux and amount of SO_2_ absorbed by nanofluids

| **SiO_2_-water NF** | | **ZnO-water NF** | | **Al_2_O_3_-water NF** | | Theoretical number of bubbles (eq. (2)) | **C:**  $\tau$  **(s)** | **B:**  $\omega$ **(wt.%)** | **A:**  *d_o_*  **(mm)** | **Run No.** |
| --- | --- | --- | --- | --- | --- | --- | --- | --- | --- | --- |
| **N_avg×10_^6^**  **mol/m^2^.s** | **C­_Bulk,_**  **mol/m^3^** | **N_avg×10_^6^**  **mol/m^2^.s** | **C­_Bulk,_**  **mol/m^3^** | **N_avg×10_^6^**  **mol/m^2^.s** | **C­_Bulk,_**  **mol/m^3^** |  |  |  |  |  |
| 24±0.31 | 3.320 | 67.4±0.72 | 9.3209 | 33.6±0.39 | 4.648 | 1833 | 450 | 0.055 | 0.46 | 1 |
| 37.7±0.94 | 1.162 | 226±2.92 | 6.9626 | 40.4±0.96 | 1.245 | 153 | 300 | 0.055 | 0.57 | 2 |
| 162±4.34 | 1.245 | 510±9.64 | 3.9305 | 172±4.47 | 1.328 | 76 | 150 | 0.100 | 0.57 | 3 |
| 41.9±0.59 | 2.905 | 87.5±1.04 | 6.0642 | 57.5±0.74 | 3.984 | 229 | 450 | 0.100 | 0.57 | 4 |
| 51.2±1.03 | 1.577 | 197±2.58 | 6.0642 | 37.7±0.94 | 1.162 | 153 | 300 | 0.055 | 0.57 | 5 |
| 102±1.65 | 2.739 | 188±2.68 | 5.0535 | 83.3±1.45 | 2.241 | 121 | 300 | 0.010 | 0.68 | 6 |
| 247±6.27 | 1.660 | 535±11.5 | 3.5936 | 309±7.32 | 2.075 | 61 | 150 | 0.055 | 0.68 | 7 |
| 59.4±1.78 | 0.913 | 278±3.75 | 4.2674 | 91.8±1.98 | 1.411 | 611 | 150 | 0.055 | 0.46 | 8 |
| 86.2±3.60 | 0.664 | 423±8.19 | 3.2567 | 97±3.69 | 0.747 | 76 | 150 | 0.010 | 0.57 | 9 |
| 68.6±0.90 | 4.150 | 139±1.67 | 8.4225 | 71.3±0.93 | 4.316 | 182 | 450 | 0.055 | 0.68 | 10 |
| 37.8±0.57 | 2.324 | 84±0.98 | 5.1658 | 50±0.67 | 3.071 | 1222 | 300 | 0.100 | 0.46 | 11 |
| 33.5±0.52 | 2.324 | 109±1.26 | 7.5241 | 32.3±0.51 | 2.241 | 229 | 450 | 0.010 | 0.57 | 12 |
| 25.7±0.49 | 1.577 | 98.6±1.12 | 6.0642 | 32.4±0.53 | 1.992 | 1222 | 300 | 0.010 | 0.46 | 13 |
| 45.8±0.99 | 1.411 | 211±2.75 | 6.5134 | 35±0.92 | 1.079 | 153 | 300 | 0.055 | 0.57 | 14 |
| 89.5±1.52 | 2.407 | 100±1.63 | 2.6952 | 130±1.97 | 3.486 | 121 | 300 | 0.100 | 0.68 | 15 |

**Uncertainty analysis was performed for molar flux in tables S-3 and S-4. Uncertainties of the measured variables are listed in the follow.

| $\omega_{C_{RO_{2}}}=0.0251\frac{mol}{m^{3}}$ | $\omega_{\theta}=1 sec$ | $\omega_{r_{0}}=0.001m$ | $\omega_{V}=1e-6 m^{3}$ | $\omega_{n}=1bubble$ |
| --- | --- | --- | --- | --- |

Uncertainties of the calculated parameters were obtained from :

$$R=R\left( v_{1}, v_{2}, v_{3}, \ldots, v_{n} \right)$$

$$\frac{\omega_{R}}{R}=\sqrt{\left( \frac{\partial R}{\partial v_{1}}\frac{\omega_{1}}{R} \right)^{2}+\left( \frac{\partial R}{\partial v_{2}}\frac{\omega_{2}}{R} \right)^{2}+\left( \frac{\partial R}{\partial v_{3}}\frac{\omega_{3}}{R} \right)^{2}+\ldots+\left( \frac{\partial R}{\partial v_{n}}\frac{\omega_{n}}{R} \right)^{2}}$$

Therefore: $R=R\pm\omega_{R}$


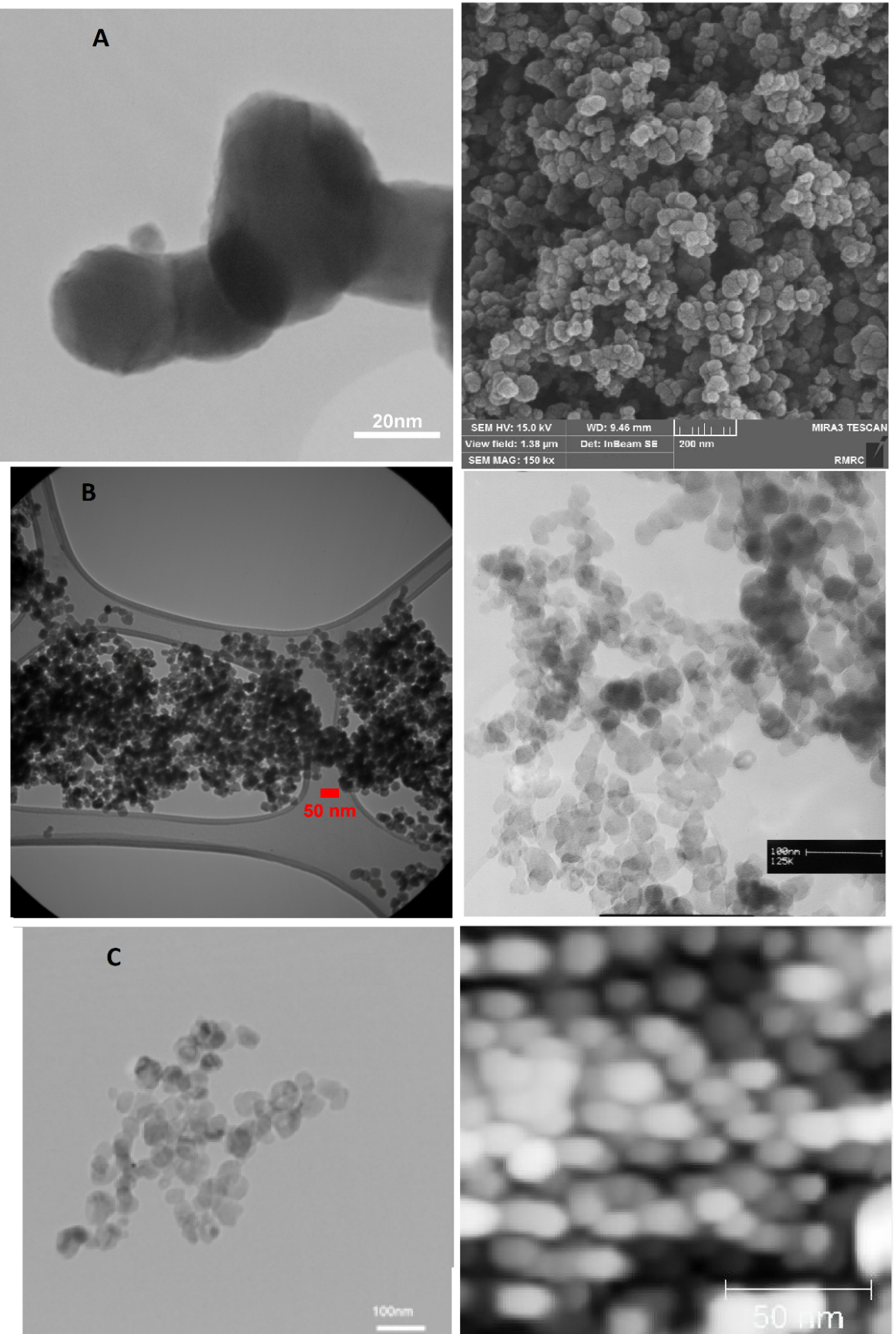


**Figure S-1.** TEM and SEM images of the (a) ZnO, (b) Al_2_O_3_,and (c) SiO_2_ NPs.


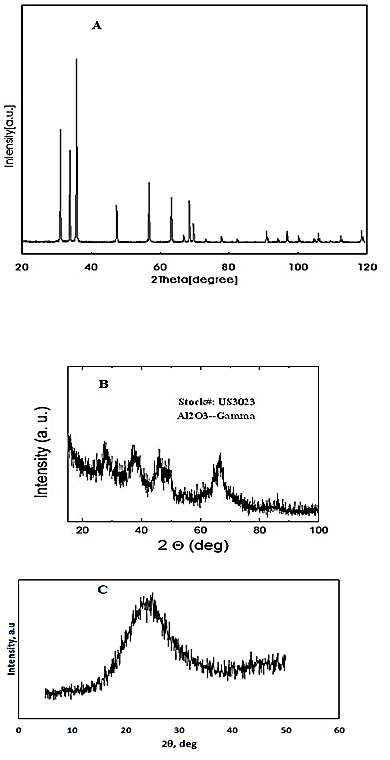


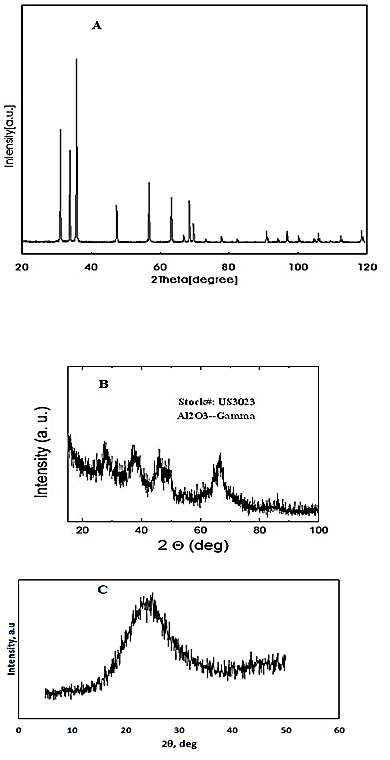

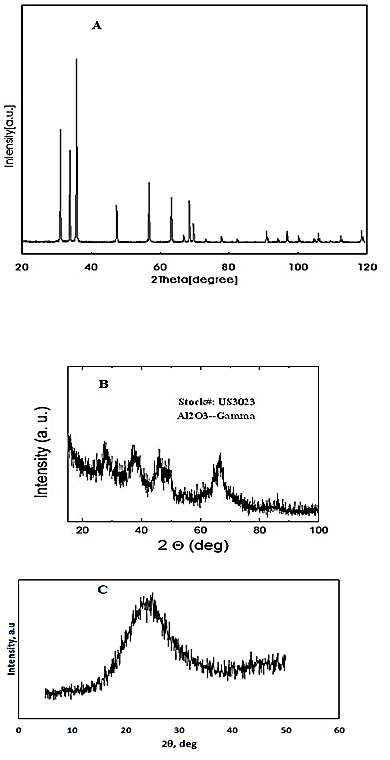


**Figure S-2.** X-Ray diffraction (XRD) analyses for the (a) ZnO, (b) Al_2_O_3_, and (c) SiO_2_ NPs.


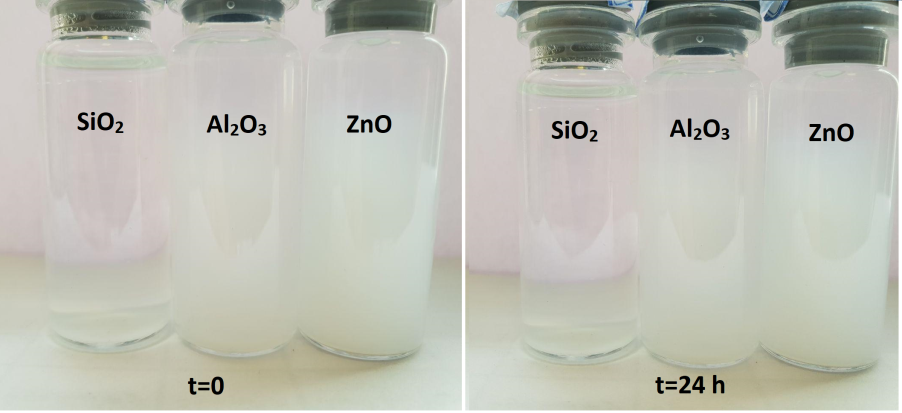


**Figure S-3.** Images of nanofluid samples during a 24-hour period to check their stability


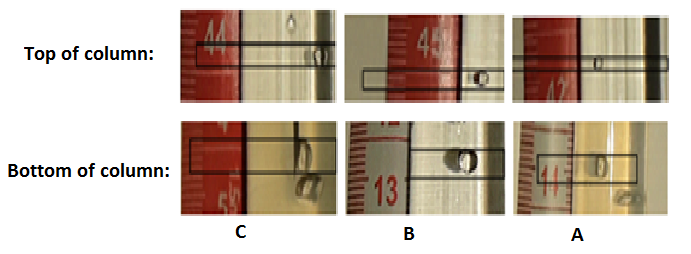


**Figure S-4.** Photos of a CO_2_ gas bubble passing into the base fluid (water) from bottom to top of the column for various nozzle sizes of (A) 0.46 mm, (B) 0.57 mm, and (C) 0.68 mm.


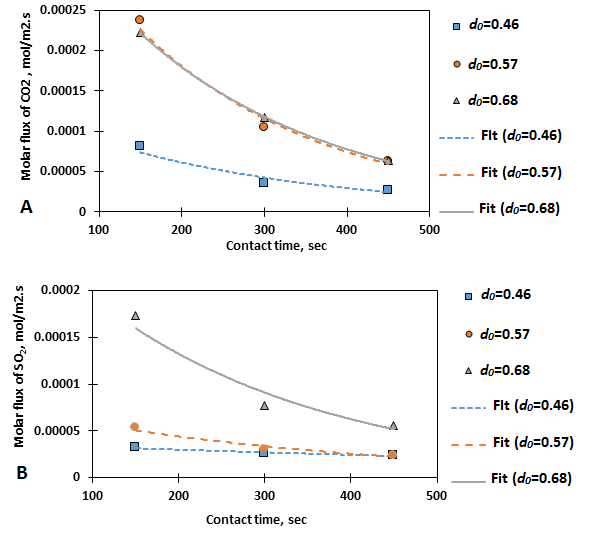


**Figure S-5.** Molar flux vs. contact time for (A) CO_2_ and (B) SO_2_ absorption in DM water


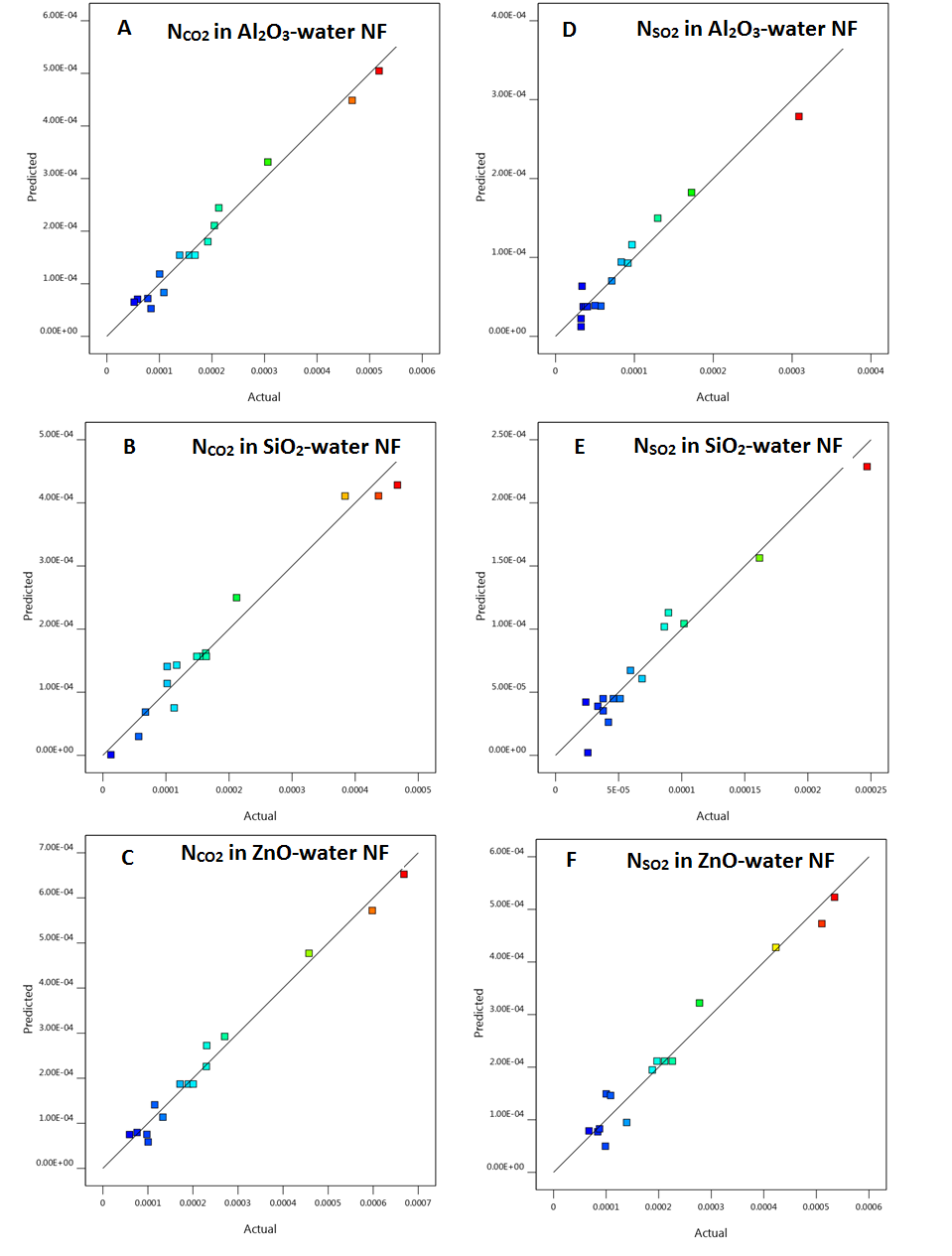


**Figure** **S-6**. Predicted vs. actual values for the molar fluxes of CO_2_ (A to C) and SO_2_ (D to F) into the various nanofluids


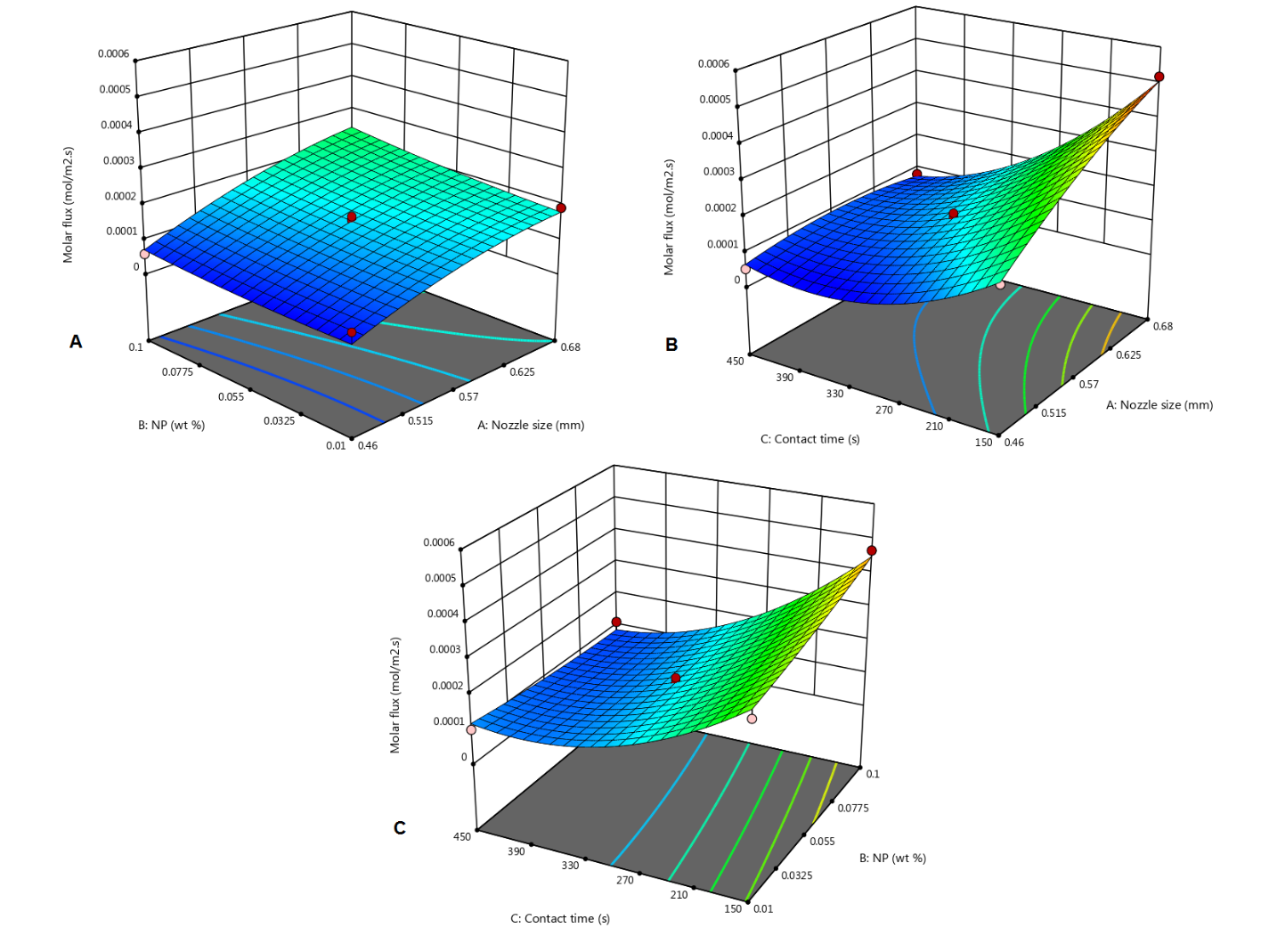


**Figure S-7.** Effect of parameters on molar flux of CO_2_ in Al_2_O_3_-water nanofluid: (A) *d_o_* and $\omega$, (B) *d_o_* and $\tau$, (C) $\omega$ and $\tau$.


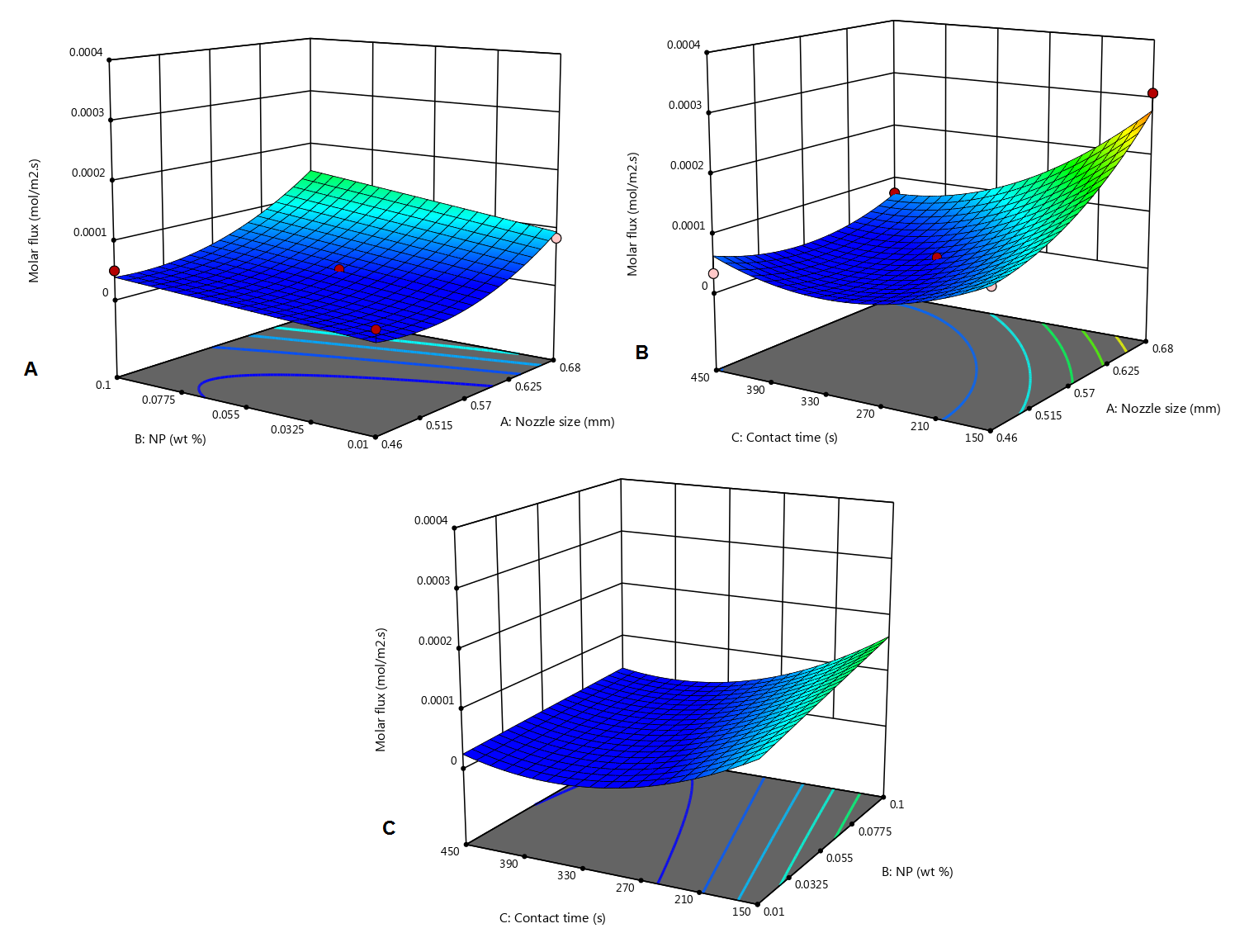


**Figure S-8.** Effect of parameters on molar flux of SO_2_ in Al_2_O_3_-water nanofluid: (A) *d_o_* and $\omega$, (B) *d_o_* and $\tau$, (C) $\omega$ and $\tau$.


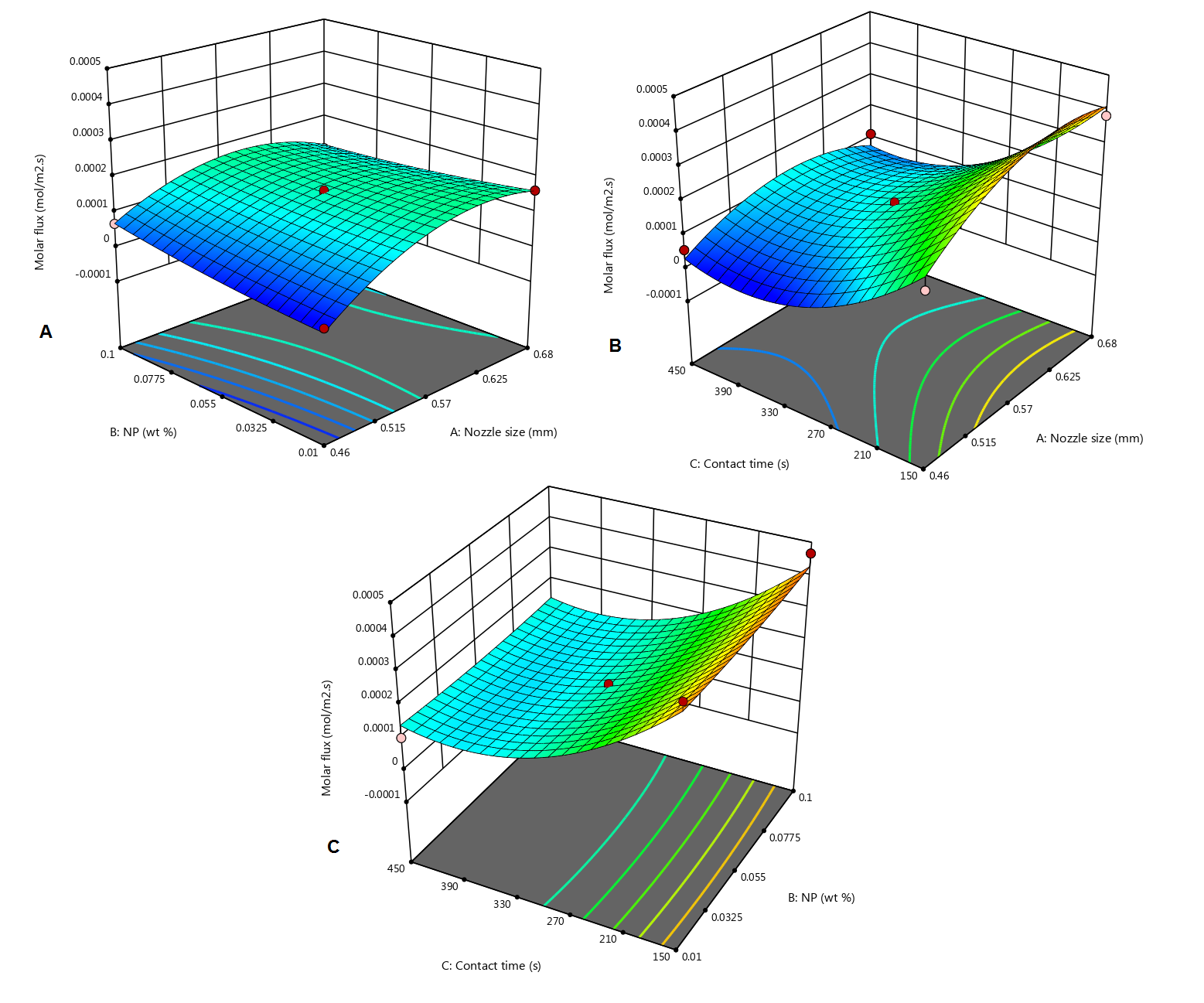


**Figure S-9.** Effect of parameters on molar flux of CO_2_ in SiO_2_-water nanofluid: (A) *d_o_* and $\omega$, (B) *d_o_* and $\tau$, (C) $\omega$ and $\tau$.


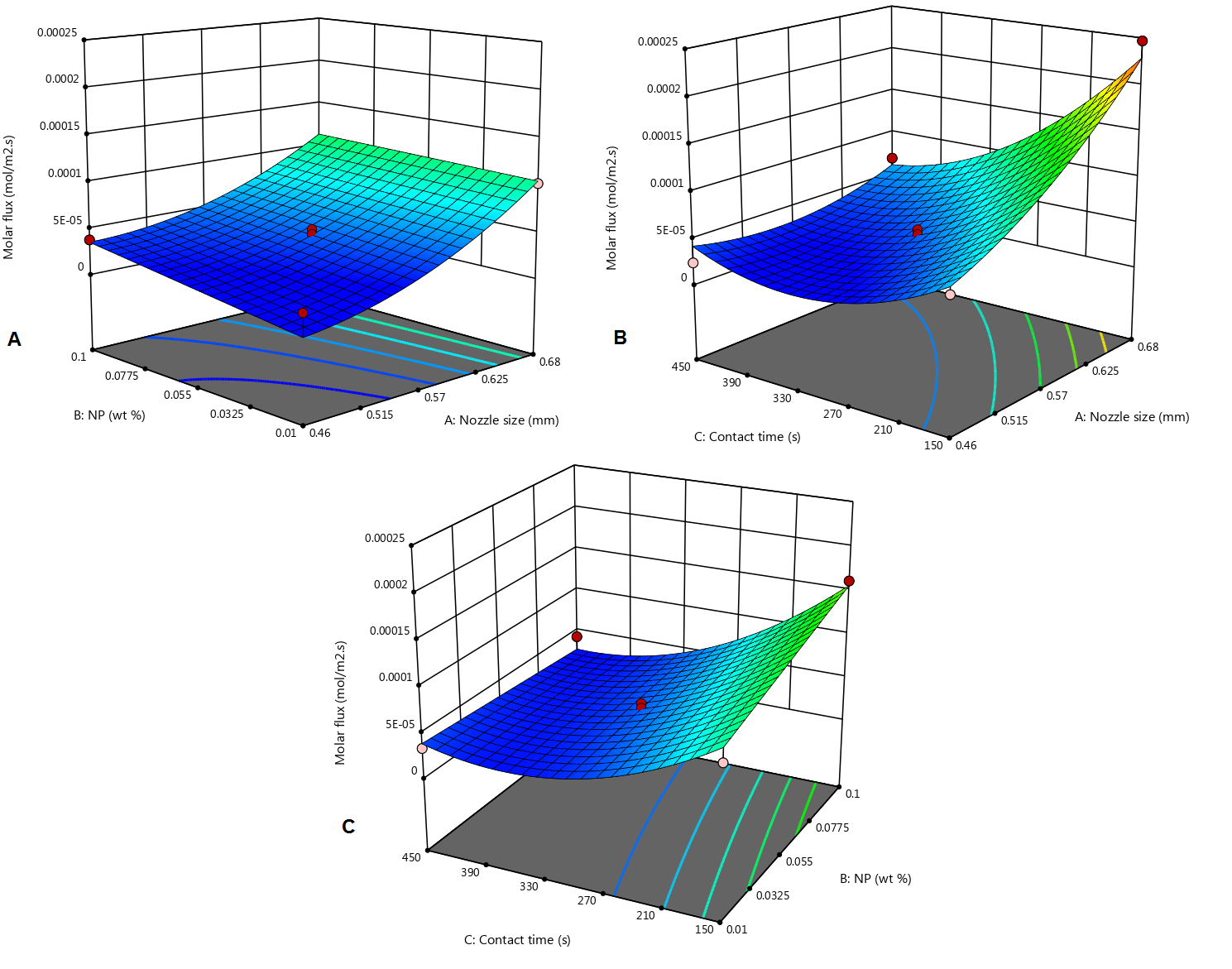


**Figure S-10.** Effect of parameters on molar flux of SO_2_ in SiO_2_-water nanofluid: (A) *d_o_* and $\omega$, (B) *d_o_* and $\tau$, (C) $\omega$ and $\tau$.
